# Shared decision-making interventions in neuro-oncology practice: a systematic review

**DOI:** 10.1007/s11060-025-05141-7

**Published:** 2025-06-23

**Authors:** Jasmine G. Hughes, Francesca M. Cozzi, Veronica Phillips, Stephen J. Price

**Affiliations:** 1 Cambridge Brain Tumour Imaging Laboratory, Division of Neurosurgery, Department of Clinical Neurosciences, Addenbrooke’s Hospital, University of Cambridge, Cambridge, UK; 2 University of Cambridge Medical Library, University of Cambridge, Cambridge, UK; 3 Department of Clinical Neurosciences Cambridge, University of Cambridge, Cambridge, UK

**Keywords:** Shared decision-making, Neuro-oncology, Neurosurgery

## Abstract

**Background:**

Shared decision-making (SDM) has been shown to be beneficial to patients and improve health outcomes. While more research is being conducted on the topic of SDM, the incorporation of interventions to facilitate or improve SDM in neuro-oncology has not been widely studied. This study aimed to systematically review the types and impact of SDM interventions used in neuro-oncology.

**Methods:**

Systematic searches were conducted in Medline, Embase, Global Health, Cinahl, Web of Science, and Scopus from inception to May 2024. Full-text, peer-reviewed articles were evaluated based on inclusion criteria. Data extracted from articles included the author, year, location, type of intervention, and variable outcomes.

**Results:**

The searches resulted in 4674 original articles. Four studies with a total of 172 patients diagnosed with anaplastic oligodendroglioma, anaplastic astrocytoma, high-grade glioma (HGG), low-grade glioma (LGG), glioblastoma, and brain metastases met the inclusion criteria. Types of SDM interventions included SDM training for health care workers, decision grids, three-dimensional (3D) printed models of brain tumors, goals of care videos, and an online tool providing information on disease progression. Overall impact of SDM interventions resulted in improvement in patients’ understanding of their medical condition, treatment options, and satisfaction with the SDM process.

**Conclusion:**

SDM can be improved through the use of interventions and aids and can have a positive impact on brain tumor patients. However, there is a significant gap within neuro-oncology literature on SDM interventions. Therefore, to understand how to best improve SDM from the perspective of patients, there is a pressing need for more research on SDM interventions in neuro-oncology.

**Supplementary Information:**

The online version contains supplementary material available at 10.1007/s11060-025-05141-7.

## Introduction

In clinical practice, shared decision-making (SDM) is the balance between a physician’s experience and expertise and a patient’s individual perspectives and preferences for care [1]. One of the four pillars of medical ethics is autonomy, which describes the respect that medical practitioners should have for a patient’s right to self-determination and informed decision-making [2]. This is taken into consideration with the other three pillars: beneficence, non-maleficence, and justice [2]. Beneficence and non-maleficence, in particular, speak to a physician’s commitment to doing good for a patient and, thereby, doing no harm [2]. The concept of medical paternalism is now antiquated, whereas a physician should not outrightly override a patient’s autonomy if that patient has the capacity for informed decision-making.

While the pendulum should not swing to either extreme of medical paternalism or sole patient-driven decision-making, there should be a harmonic balance that involves the patient in healthcare decisions that are guided by a physician’s expertise; in so doing, a physician considers a patient’s perspectives, goals of care, culture, and religious preferences. This partnership between physician and patient is the cornerstone of SDM, particularly for neuro-oncological illnesses, such as malignant brain tumors. Despite certain limitations to SDM in neuro-oncology, such as complexities with patients’ waning decision-making capacity over time, SDM may also be inclusive of family members or friends whom the patient stipulates as proxies. Nonetheless, the pivot away from exclusive medical paternalism has been due, in part, to the benefits that SDM can offer. These benefits are multifaceted, namely reduction in patient uncertainty and decisional conflict; improved communication between patient and physician, which may foster better rapport and trust; and patients’ improved emotional well-being [3]. 

Given that neuro-oncological patients are especially vulnerable to difficult decisions that may pertain to functional and cognitive decline, as well as dismal overall prognosis, SDM is crucial. SDM cultivates a caregiving approach that is guided by both a physician’s expertise and a physician’s respect for patient autonomy. In so doing, the patient is not a mere object of care, but rather a partner in it. With this in mind, we systematically reviewed the literature to understand the impact of implementing SDM interventions for brain tumor patients specifically, while also uncovering some of the challenges involved in that process.

## Methods

The systematic review was constructed based on the PRISMA (Preferred Reporting Items for Systematic Reviews and Meta-Analyses) [4] checklist and registered on PROSPERO (CRD42024608686).

### Search strategy and information sources

Medline (via Ovid), Embase (via Ovid), Global Health (via Ebscohost), Cinahl (via Ebscohost), Web of Science (Core Collection), and Scopus are six databases that were searched from inception to May 2024 by V.P. In addition, the Global Index Medicus and Overton were searched from inception to May 2024 to identify grey literature not captured in the database searches. The search strategy was peer-reviewed by two librarians using the Peer Review of Electronic Search Strategies (PRESS) checklist [5] and the strategy was evaluated against the PRISMA-S guidelines [6]. Databases were searched by V.P. separately, rather than multiple databases being searched on the same platform. The search syntax was adapted for each database to account for variation between thesaurus terms/controlled vocabulary across databases. Results were imported to EndNote 21 by V.P. for deduplication, using the method outlined by Bramer et al. [7]. The searches were all rerun before submission to include any papers published between the initial search and submission for peer review. Dates when searches were run and full search strategies used in each database are documented in the supplemental materials.

### Inclusion and exclusion criteria

Studies were considered eligible if they met the following inclusion criteria: full-text, published, peer-reviewed empirical studies with a patient population of adults (18 years and older) diagnosed with a brain tumor, with the exploration of the use of a shared decision-making intervention. Studies that discuss SDM without the use of an intervention and reported outcomes from patients were excluded. Non-English manuscripts, abstracts only, and conference proceedings were excluded.

### Study selection and data extraction

Titles and abstracts were screened on the Covidence (Covidence systematic review software, Veritas Health Innovation) for subsequent full-text review for those included after this screening. A template for data extraction was used for selected studies, which included the following information: author, year, institution, study design, participants, intervention, treatment options, and variable patient-reported outcomes. Data extraction was completed independently by JGH and FMC. After consensus was reached, the extracted data sheets were assessed by SJP.

### Narrative synthesis

We performed a narrative synthesis based on the objective of the systematic review. The synthesis consists of two parts: (i) exploring the type of SDM intervention used in the studies and (ii) assessing the impact of the SDM intervention. A minimum of two studies were required for the narrative synthesis.

### Quality assessment

The validity and methodology quality of articles were assessed with a template based on the Mixed Methods Appraisal Tool (MMAT) [8–10]. The MMAT is a tool to appraise multiple types of study designs, including quantitative, qualitative, and mixed methods [8–10]. The criteria for each study design category were determined to be either “yes”, “no”, or “can’t tell”. JGH and FMC completed the quality assessment individually, and a consensus was obtained. Quality assessments provided a review of the methodology quality of included studies and required no further exclusions.

## Results

### Search results

**Fig. 1:**
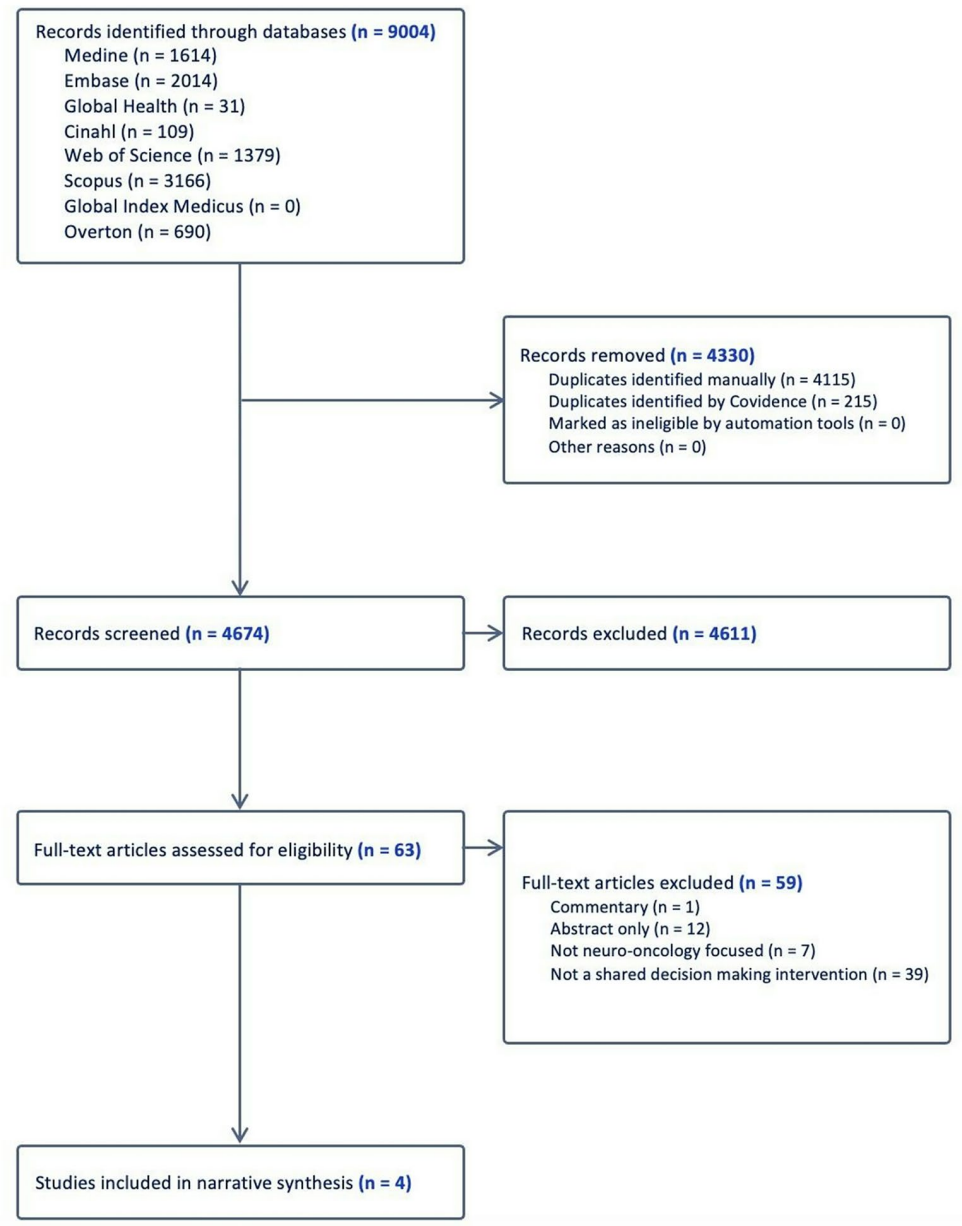
PRISMA (Preferred Reporting Items for Systematic Reviews and Meta-Analyses) Flowchart

The systematic search across all databases resulted in 9004 records. After the removal of duplicates in EndNote, a total of 4889 records were imported into Covidence. Further duplicates were identified and removed in Covidence, which resulted in 4674 original articles. The screening and selection processes are shown in the PRISMA flowchart (Fig. 1).

### Design and characteristics of included studies

Four studies with a total of 172 patients, with diagnoses including anaplastic oligodendroglioma, anaplastic astrocytoma, high-grade glioma (HGG), low-grade glioma (LGG), unspecified glioma, glioblastoma, and brain metastases, met the inclusion criteria (Table 1**)**.

**Table 1:** Characteristics of included studies

Author	El-Jawahri et al. 2010 [11]	van de Belt et al. 2018 [12]	van Diest et al. 2022 [13]	Leu et al. 2023 [14]
Location	USA	Netherlands	Netherlands	United Kingdom
Study Design	Quantitative randomized controlled trial	Qualitative individual interviews	Qualitative patient-proxy interviews	Quantitative nonrandomized
Objective	To determine the impact of a goals of care video on decision-making	To understand the value of three-dimensional printed brain tumor models	To explore the use of an online tool for advanced care planning	To assess the impact of SDM on patient and staff satisfaction
Number of Participants	27 patients in the control narrative group; 23 patients in the video intervention group	11 patients interviewed	15 patients and their proxies were interviewed	22 patients in Phase 1 (before SDM implemented); 74 patients in Phase 2 (SDM as standard of care)
Type of Brain Tumor(s)	Malignant glioma (Glioblastoma, Anaplastic astrocytoma, Anaplastic oligodendroglioma)	Glioma (Grade 2, 3, and unknown)	Glioblastoma	Intracranial glioma (HGG, LGG) and metastases
Intervention	Video-based decision aid	Three-dimensional brain tumor models	Glioblastoma progression-based online tool	SDM training for medical staff; decision grids
Decisions	Preferred level of care	Surgical treatment options	Advance care planning	Treatment options
Outcomes	Preference for level of care and CPR; knowledge assessment; Decisional Conflict Scale	Experience of positive and negative aspects of the model	Experience of quality of life and uncertainty	Patient and Health Staff Satisfaction

In the RCT study conducted by El-Jawahri et al. [11] the impact of a goals in care video on decision-making in comparison to a standard verbal narrative was assessed. Patients with malignant glioma were randomized into two groups: a control group of 27 patients who were only given a verbal narrative and an intervention group of 23 patients who were provided with the video supplement [11]. Outcome measures included a cardiopulmonary resuscitation (CPR) preference assessment, knowledge assessment, the uncertain subset within the Decisional Conflict Scale, and a post-intervention preference for care assessment based on four categories: life-prolonging care, comfort care, basic care, and uncertain [11].

The single arm study by van de Belt and colleagues [12] focused on understanding the value of 3D-printed brain tumor models. A total of 11 adult patients with glioma who completed magnetic resonance imaging (MRI) and diffusion tensor imaging (DTI) were all given a full-scale 3D model of their specific brain tumor [12]. To determine the facilitators, barriers, and effects of the 3D models, semi-structured interviews were conducted with patients [12]. 

The qualitative study by van Diest et al. [13] explored the use of an online tool on the experiences of patient-proxy dyads. Only adult patients with a confirmed diagnosis of glioblastoma and their proxies were included in the study [13]. The online tool was constructed with the aim of informing patients of the progression of the disease. A total of 15 patient-proxy dyads were provided the online tool without instructions during “think-aloud sessions” [13]. Outcome data were collected by semi-structured interviews from a non-blinded research team [13].

The prospective study by Leu et al. [14] assessed the impact of SDM processes on satisfaction of both patients and medical staff. The patient cohort was divided into two groups of patients with intracranial glioma and metastases who were undergoing treatment considerations [14]. The first group consisted of 22 patients before the introduction of SDM, and the second group consisted of 74 patients after the implementation of SDM [14]. The implementation of SDM included training for medical staff and the use of decision grids tailored for three types of intracranial tumors. Satisfaction for patients was measured using the CollaboRATE questionnaire [15–18] and the Advancing Quality Alliance (AQuA) questionnaire [19] for medical staff.

### Methodology quality assessment

All included studies demonstrated a clear research question, and the collected data from each study enabled the research questions to be answered. The methodology quality assessment for all four included studies is shown in Table 2.

**Table 2:** Methodology quality assessment of included studies

Category of study designs	Methodological quality criteria	El-Jawahri et al. 2010	van de Belt et al. 2018	van Diest et al. 2022	Leu et al. 2023
Screening questions	S1. Are there clear research questions?	+	+	+	+
	S2. Do the collected data allow to address the research questions?	+	+	+	+
1. Qualitative	1.1. Is the qualitative approach appropriate to answer the research question?	N/A	+	+	N/A
	1.2. Are the qualitative data collection methods adequate to address the research question?	N/A	+	+	N/A
	1.3. Are the findings adequately derived from the data?	N/A	+	+	N/A
	1.4. Is the interpretation of results sufficiently substantiated by data?	N/A	+	+	N/A
	1.5. Is there coherence between qualitative data sources, collection, analysis and interpretation?	N/A	+	+	N/A
2. Quantitative randomized controlled trials	2.1. Is randomization appropriately performed?	+	N/A	N/A	N/A
	2.2. Are the groups comparable at baseline?	---	N/A	N/A	N/A
	2.3. Are there complete outcome data?	+	N/A	N/A	N/A
	2.4. Are outcome assessors blinded to the intervention provided?	---	N/A	N/A	N/A
	2.5 Did the participants adhere to the assigned intervention?	+	N/A	N/A	N/A
3. Quantitative nonrandomized	3.1. Are the participants representative of the target population?	N/A	N/A	N/A	?
	3.2. Are measurements appropriate regarding both the outcome and intervention (or exposure)?	N/A	N/A	N/A	+
	3.3. Are there complete outcome data?	N/A	N/A	N/A	---
	3.4. Are the confounders accounted for in the design and analysis?	N/A	N/A	N/A	---
	3.5. During the study period, is the intervention administered (or exposure occurred) as intended?	N/A	N/A	N/A	+
4. Quantitative descriptive	4.1. Is the sampling strategy relevant to address the research question?	N/A	N/A	N/A	N/A
	4.2. Is the sample representative of the target population?	N/A	N/A	N/A	N/A
	4.3. Are the measurements appropriate?	N/A	N/A	N/A	N/A
	4.4. Is the risk of nonresponse bias low?	N/A	N/A	N/A	N/A
	4.5. Is the statistical analysis appropriate to answer the research question?	N/A	N/A	N/A	N/A
5. Mixed methods	5.1. Is there an adequate rationale for using a mixed methods design to address the research question?	N/A	N/A	N/A	N/A
	5.2. Are the different components of the study effectively integrated to answer the research question?	N/A	N/A	N/A	N/A
	5.3. Are the outputs of the integration of qualitative and quantitative components adequately interpreted?	N/A	N/A	N/A	N/A
	5.4. Are divergences and inconsistencies between quantitative and qualitative results adequately addressed?	N/A	N/A	N/A	N/A
	5.5. Do the different components of the study adhere to the quality criteria of each tradition of the methods involved?	N/A	N/A	N/A	N/A
Score		5/7	7/7	7/7	4/7

Questions not applicable for study designs are marked N/A, not applicable; --, no; +, yes;?, can’t tell

In the study conducted by El-Jawahri et al. [11] a computer-based randomization scheme assigned participants into groups. Baseline characteristics in each group were not comparable, as the intervention group had a higher overall mean age, percentage of male participants, and percentage of participants with an “excellent/very good/good” health status compared to the control group. The study’s assigned intervention was adhered to and completed by all participants. As the outcome assessors were not blinded to assigned interventions, there is a high risk of bias within the study.

Van de Belt et al.’s [12] exploratory approach with semi-structured interviews and the data collection method of audio-recording the interviews and transcribing verbatim audio transcripts, and reaching thematic saturation, was appropriate to understand barriers and facilitators of using a 3D brain tumor model. The study’s findings of participants’ experience with the model were adequately derived and interpreted from the data using existing frameworks to classify barriers, facilitators, and effects. There was coherence between the qualitative sources of participants’ interviews and the research team’s data collection, analysis, and interpretation.

The qualitative approach of the study by van Diest et al. [13] using think-aloud sessions and semi-structured interviews, which were audio-recorded and transcribed verbatim in two phases to reach data saturation, was appropriate to determine patients’ and their proxies’ experiences with using the online tool. Data findings based on patient-proxy dyads’ experiences with the online tool were adequately derived and interpreted using thematic analysis. The patient-proxy dyads’ interviews and the research team’s data collection, analysis, and interpretation were all coherent.

In the assessment of Leu et al.’s study [14] it is unclear if participants were a comprehensive representation of the glioma patient population, as the only demographic data provided are the participants’ age (range and mean age) and glioma type. The CollaboRATE and AQuA questionnaires were appropriate outcome measurements for patient and staff satisfaction. While all patient participants completed the CollaboRATE questionnaire, one staff participant did not complete the AQuA after the introduction of the SDM intervention. The customized decision grids were not validated prior to use in the study and were not accounted for as a confounder in the study design and analysis. The SDM intervention was implemented as intended in the study for all participants.

### Narrative synthesis of interventions

#### SDM Training for Medical Professionals

In the study by Leu et al. [14] one lead neurosurgeon was provided an SDM training by National Health Service (NHS) England, which consisted of traditional classroom teaching, discussions, role playing, and patients’ presentations over a period of two days. After completion of the course, the medical staff were cascade-trained with a foundation in SDM and the techniques to use as standard care by the lead neurosurgeon. The exact details of the SDM training course were not provided. After incorporating SDM as a standard care practice, the medical staff’s satisfaction significantly improved from 61.68 to 90.95% on the AQuA questionnaire [14].

#### Decision grids

Decision grids for three types of intracranial tumors (HGG, LGG, metastases) developed by Leu et al. [14] were based on the National Institute for Health and Care Excellence (NICE) guidelines [20] and an Option Grid template [21]. Each decision grid was composed of specific treatment options based on the type of tumor. Treatment options provided in grids included Best Medical Care (BMC), resection and biopsy for HGG patients; active surveillance, resection, and biopsy for LGG patients; BMC, stereotactic radiosurgery or radiotherapy, and resection for patients with metastases. Benefits and risks (adjusted based on individual patients’ profiles) were provided for each treatment option in the grids. However, the use of decision grids did not result in a significant improvement in patients’ CollaboRATE questionnaire scores [14]. As the decision grids were used in combination with SDM training of medical staff in the study, it is unclear how the grids can directly impact the process of facilitating the SDM for brain tumor patients.

#### 3D brain tumor model

 As mentioned in van de Belt et al.‘s study [ 12 ] 3D models of specific patients’ brain tumors were constructed using an Ultimaker 3D printer with imported functional MRI and DTI images from patients’ imaging scans. Segmented parts were printed in particular colors to identify certain regions of the brain model, including the tumor, corticospinal tract, areas for speech, language, and motor processing. The associate costs for each 3D model were less than $6 USD, but the cost of the 3D printer used was approximately $1741 USD [ 12 ]. In semi-structured interviews, patients reported that the 3D models provided positive effects of improved communication, coping, acceptance, knowledge, and understanding of risks and benefits. In addition, emotional confrontation was reported as a negative effect [ 12 ]. 

#### Glioblastoma progression online tool

An online tool that covers the progression of glioblastoma was designed for adult patients and their proxies within the study by van Diest et al. [13]. The online tool provided information on possible symptoms (initial, during recurrent growth, end-of-life phase), anti-tumor treatment, and palliative treatment. In addition, a line plot of tumor size over time was provided with interrupted lines indicating unpredictability in determining the amount of time for tumor recurrence and end-of-life stage. The Phase 1 group mentioned that the content of the online tool provided positive aspects of useful information with a clear overview [13]. Negative aspects that the Phase 1 group mentioned included lengthy, unclear medical jargon and missing information [13]. After revisions to the online tool based on the Phase 1 group’s feedback were implemented, the Phase 2 group reported that information on treatment options for tumor relapse provided hope, and they valued the topic of psychological aspects [13].

#### Goals of care video

A video supplement was created by El-Jawahri et al. [11] to depict three levels of care for malignant glioma patients in Boston, USA. The six-minute video provided images and scenes for each type of care using a cinema vérité style. In the control group, the participants’ average age was 56 with a range from 47 to 77 years old. The video intervention group had a slightly younger participants’ age average of 51 with a range of 32–72 years old. The implementation of the video resulted in a higher preference for comfort care (91.3%, *n* = 21) compared to the control group (22.2%, *n* = 6).^11^ A higher knowledge assessment score (5.3) was seen with the use of the video intervention compared to the control group (4.6) [11]. Patients expressed a significantly lower willingness to undergo CPR in the video intervention group (8.7%, *n* = 2) in comparison to the control group (40.7%, *n* = 11).^11^ The majority of participants (82.6%) reported being “very comfortable” with the video and would “definitely recommend” it to other cancer patients (82.6%).^11^

## Discussion

In this systematic review, the current literature on SDM interventions in neuro-oncology was assessed. A sparse implementation of SDM interventions was apparent in neuro-oncology practice settings, and most studies primarily focus on treating glioma patients. While a range of methodologies, including qualitative and RCTs, were used, all studies’ outcomes included patients’ perspectives of the impact of each SDM intervention.

The positive impact of individually customized interventions for patients, such as decision grids and 3D tumor models, was noted. While patients can have the same type of brain cancer, the tumor aggressiveness and progression can vary and require different combinations of treatments. Providing each patient with a customized SDM intervention during their initial neurosurgical consultation can provide an increase in the understanding of their specific tumor, as well as the risks and benefits of each treatment option tailored for them.

Patients with advanced brain cancer can have significant cognitive decline, which can impair their decision-making capacity. The consideration of cognitive deficits within brain tumor patients was not included in most studies in this review, as some patients had to demonstrate decision-making capacity to be included in these studies. This was seen with the inclusion criteria of a Folstein Mini-Mental Status Examination (MMSE) [22] score greater than or equal to 24 in the El-Jawahri et al. study [11]. However, before patients develop cognitive impairments, they can designate a proxy who can decide treatments on their behalf. Only the van Diest et al. [13] study included proxies in the SDM intervention implemented.

Establishing SDM as the standard of care for patients must involve providing SDM training for medical staff, as included in the Leu et al. [14] study. Institutions such as the NHS and the Massachusetts General Hospital (MGH) Health Decision Science Center have created SDM training courses that provide a foundation in the components of SDM and how to best implement SDM in practice [23, 24]. However, the implementation of SDM that patients can receive can still vary, as different countries have different legal policies and frameworks that govern how SDM can be embedded into clinical practices.

In comparison to other type of cancers and specialties, the literature on SDM interventions usage in neuro-oncology seems to lag behind. Well-documented interventions in cardiology practices, such as clinician training, nurse-led coaching, and decision aids (web-based, online-based, audio-based, and printed) to improve SDM in patients with atrial fibrillation and coronary artery disease have resulted in reduced decisional conflict [25–30]. In uro-oncology, decision aids structured in a variety of formats have been used to provide guidance on personalized treatment options for prostate, kidney, and bladder cancer patients [31–37]. In addition, SDM decision aids have improved patient-provider communication and satisfaction among patients considering screening for breast, colorectal, and bowel cancer [38–43]. 

To improve the understanding of SDM interventions in neuro-oncology, future directions should aim to uncover the impact of SDM in patients with recurrent high-grade tumors and their understanding of potential changes to quality of life with certain treatments. As more aggressive treatments can lead to a decline in quality of life for patients, it is essential for patients to understand possible health outcomes and their preferences to be incorporated into their treatment plan.

### Limitations

There are a few limitations to note within this review. The small size of eligible articles, small sample sizes, and variability in study methodologies can contribute to low generalizability and statistical relevance to the overall brain tumor patient population. Another limitation is the locations of the studies, which were either conducted in the United Kingdom, the United States, or the Netherlands. Given the potential bias from being solely from a High-Income Countries (HICs) perspective, the overall impact of certain SDM interventions may not be applicable within Low-and-Middle-Income Countries (LMICs) settings. As only articles written in English were included in this review, literature covering this topic in other languages may not have been discussed.

## Conclusion

This review resulted in a variety of interventions, which were shown to have a positive impact on SDM for brain tumor patients. However, this systematic review highlights the current sparsity of SDM interventions mentioned in neuro-oncology literature. Therefore, future research on SDM interventions in neuro-oncology is urgently warranted.

## Electronic supplementary material

Below is the link to the electronic supplementary material.

Supplementary Material 1

Supplementary Material 2

## Data Availability

No datasets were generated or analysed during the current study.

## References

[1] von Sorensen H (2025) Shared decision-making in neuro-oncology: existing practices and future steps. Neurooncol Pract 12(2):179–180. 10.1093/nop/npaf005 40110056 10.1093/nop/npaf005 PMC11913641

[2] Shetty N (2023) Medical ethics and law. Indian J Orthop 57(11):1744–1747. 10.1007/s43465-023-00972-w 37881274 10.1007/s43465-023-00972-w PMC10593724

[3] von Sorensen H, Piil K, Dahl Steffensen K, Rom Poulsen F (2020) Shared decision making in high-grade glioma patients - a systematic review. Neurooncol Pract 7(6):589–598. 10.1093/nop/npaa042 33304599 10.1093/nop/npaa042 PMC7716176

[4] Moher D, Liberati A, Tetzlaff J et al (2009) PRISMA group. Preferred reporting items for systematic reviews and Meta-Analyses: the PRISMA statement. BMJ 339:b2535 19622551 10.1136/bmj.b2535 PMC2714657

[5] McGowan J, Sampson M, Salzwedel DM, Cogo E, Foerster V, Lefebvre C (2016) PRESS peer review of electronic search strategies: 2015 guideline statement. J Clin Epidemiol 75:40–46 27005575 10.1016/j.jclinepi.2016.01.021

[6] Rethlefsen ML, Kirtley S, Waffenschmidt S, Ayala AP, Moher D, Page MJ, Koffel JB (2021) PRISMA-S: an extension to the PRISMA statement for reporting literature searches in systematic reviews. Syst Reviews 10:1–19 10.5195/jmla.2021.962 PMC8270366 34285662

[7] Bramer WM, Giustini D, De Jonge GB, Holland L, Bekhuis T (2016) De-duplication of database search results for systematic reviews in endnote. J Med Libr Assoc 104:240–243 27366130 10.3163/1536-5050.104.3.014 PMC4915647

[8] Hong QN, Gonzalez-Reyes A, Pluye P (2018) Improving the usefulness of a tool for appraising the quality of qualitative, quantitative and mixed methods studies, the mixed methods appraisal tool (MMAT). J Eval Clin Pract 24(3):459–467 29464873 10.1111/jep.12884

[9] Hong QN, Pluye P, Fàbregues S et al (2019) Improving the content validity of the mixed methods appraisal tool: a modified e-Delphi study. J Clin Epidemiol 111:49–59e1 30905698 10.1016/j.jclinepi.2019.03.008

[10] Pluye P, Gagnon MP, Griffiths F et al (2009) A scoring system for appraising mixed methods research, and concomitantly appraising qualitative, quantitative and mixed methods primary studies in mixed studies reviews. Int J Nurs Stud 46(4):529–546 19233357 10.1016/j.ijnurstu.2009.01.009

[11] El-Jawahri A, Podgurski LM, Eichler AF et al (2010) Use of video to facilitate end-of-life discussions with patients with cancer: a randomized controlled trial. J Clin Oncol 28(2):305–310 19949010 10.1200/JCO.2009.24.7502 PMC3040012

[12] van de Belt TH, Nijmeijer H, Grim D, Engelen LJLPG, Vreeken R, van Gelder MMHJ, Ter Laan M (2018) Patient-Specific Actual-Size Three-Dimensional printed models for patient education in glioma treatment: first experiences. World Neurosurg 117:e99–e105. 10.1016/j.wneu.2018.05.190 29870846 10.1016/j.wneu.2018.05.190

[13] van Diest E, Oldenmenger WH, Eland M, Taal W (2022) Evaluation of an online tool about the expected course of disease for glioblastoma patients - A qualitative study. Neurooncol Pract 9(5):411–419. 10.1093/nop/npac033 36127891 10.1093/nop/npac033 PMC9476974

[14] Leu S, Cahill J, Grundy PL (2023) A prospective study of shared decision-making in brain tumor surgery. Acta Neurochir (Wien) 165(1):15–25. 10.1007/s00701-022-05451-z 36576561 10.1007/s00701-022-05451-z PMC9795149

[15] Barr PJ, Forcino RC, Thompson R, Ozanne EM, Arend R, Castaldo MG, O’Malley AJ, Elwyn G Evaluating collaborate in a clinical setting: analysis of mode effects on scores, response rates and costs of data collection. BMJ Open 2017 7:e014681. 10.1136/bmjopen-2016-014681 10.1136/bmjopen-2016-014681 PMC5372080 28341691

[16] Barr PJ, Thompson R, Walsh T, Grande SW, Ozanne EM, Elwyn G (2014) The psychometric properties of collaborate: a fast and frugal patient-reported measure of the shared decision-making process. J Med Internet Res 16:e2. 10.2196/jmir 24389354 PMC3906697

[17] Barr PJ, Thompson R, Walsh T, Grande SW, Ozanne EM, Elwyn G Correction: The psychometric properties of collaborate: a fast and frugal patient-reported measure of The shared decision-making process. J Med Internet Res 2015 17:e32. 10.2196/jmir.4272 10.2196/jmir.4272 PMC4353887 25667387

[18] Elwyn G, Barr PJ, Grande SW, Thompson R, Walsh T, Ozanne EM Developing collaborate: a fast and frugal patient-reported measure of shared decision making in clinical encounters. Patient Educ Couns 2013 93:102–107. 10.1016/j.pec.2013.05.009 10.1016/j.pec.2013.05.009 23768763

[19] NHS England Aqua: Advancing Quality Alliance. https://aqua.nhs.uk. Accessed 10 May 2025

[20] NICE guideline (NG99) (2018): Brain Tumours (primary) and brain metastases in adults (2018)

[21] OptionGridTM (2025) https://optiongrid.ebsco.com. Accessed 10 May

[22] Folstein MF, Whitehouse PJ (1983) Cognitive impairment of alzheimer disease. Neurobehav Toxicol Teratol 5:631–634 6366602

[23] NHS Shared Decision Making Guided Resources (2025) https://www.england.nhs.uk/personalisedcare/shared-decision-making/guidance-and-resources/. Accessed. 10 May

[24] MGH Health Decision Sciences Center (2025) Shared Decision Making Training for Clinicians. https://mghdecisionsciences.org/tools-training/clinician-training/. Accessed 10 May

[25] Case BC, Qamer SZ, Gates EM et al (2019) Shared decision making in cardiovascular disease in the outpatient setting. JACC Case Rep 1:261–270 34316804 10.1016/j.jaccas.2019.06.005 PMC8301252

[26] Coylewright M, Dick S, Zmolek B et al (2016) PCI choice decision aid for stable coronary artery disease: a randomized trial. Circ Cardiovasc Qual Outcomes 9:767–776 27803090 10.1161/CIRCOUTCOMES.116.002641

[27] Doll JA, Jones WS, Lokhnygina Y et al (2019) PREPARED study: a study of shared decision-making for coronary artery disease. Circ Cardiovasc Qual Outcomes 12:e005244 30764651 10.1161/CIRCOUTCOMES.118.005244

[28] Fraenkel L, Street RL, Towle V et al (2012) A pilot randomized controlled trial of a decision support tool to improve the quality of communication and decision-making in individuals with atrial fibrillation. J Am Geriatr Soc 60:1434–1441 22861171 10.1111/j.1532-5415.2012.04080.x PMC3419306

[29] Hess EP, Knoedler MA, Shah ND et al (2012) The chest pain choice decision aid: a randomized trial. Circ Cardiovasc Qual Outcomes 5:251–259 22496116 10.1161/CIRCOUTCOMES.111.964791

[30] Thomson RG, Eccles MP, Steen IN et al (2007) A patient decision aid to support shared decision-making on anti-thrombotic treatment of patients with atrial fibrillation: randomised controlled trial. Qual Saf Health Care 16:216–223 17545350 10.1136/qshc.2006.018481 PMC2464985

[31] Feldman-Stewart D, Tong C, Brundage MD (2018) Evaluation of a widely available patient decision aid for the treatment of prostate cancer. Patient Educ Couns 101:1761–1766 29729858 10.1016/j.pec.2018.04.015

[32] Lamers RED, Cuypers M, de Vries M, van de Poll-Franse LV, Ruud Bosch JLH, Kil PJM (2017) How do patients choose between active surveillance, radical prostatectomy, and radiotherapy? The effect of a preference-sensitive decision aid on treatment decision making for localized prostate cancer. Urol Oncol 35:37e9–3717 10.1016/j.urolonc.2016.09.007 28341494

[33] McAlpine K, Breau RH, Stacey D et al (2019) Development and acceptability testing of a patient decision aid for individuals with localized renal masses considering surgical removal with partial or radical nephrectomy. Urol Oncol 37:811e1–811e7 10.1016/j.urolonc.2019.08.014 31540831

[34] McAlpine K, Lavallée LT, Stacey D et al (2019) Development and acceptability testing of a patient decision aid for urinary diversion with radical cystectomy. J Urol 202:1001–1007 31099720 10.1097/JU.0000000000000341

[35] Berry DL, Hong F, Blonquist TM et al (2018) Decision support with the personal patient Profile-Prostate: a multicenter randomized trial. J Urol 199:89–97 28754540 10.1016/j.juro.2017.07.076 PMC5760348

[36] Pacyna JE, Kim S, Yost K et al (2018) The comparative effectiveness of decision aids in diverse populations with early stage prostate cancer: a study protocol for a cluster-randomized controlled trial in the NCI community oncology research program (NCORP), alliance A191402CD. BMC Cancer 18:788 30081846 10.1186/s12885-018-4672-3 PMC6080528

[37] Belkora J, Chan JM, Cooperberg MR et al (2020) Development and pilot evaluation of a personalized decision support intervention for low risk prostate cancer patients. Cancer Med 9:125–132 31714037 10.1002/cam4.2685 PMC6943165

[38] Miller D, Spangler J, Case D, Goff D, Singh S, Pignone M (2011) Effectiveness of a web-based colorectal cancer screening patient decision aid: a randomized controlled trial in a mixed‐literacy population. Am J Prev Med 40(6):608–615 21565651 10.1016/j.amepre.2011.02.019 PMC3480321

[39] Smith SK, Trevena L, Simpson JM, Barratt A, Nutbeam D, McCaffery KJ (2010) A decision aid to support informed choices about bowel cancer screening among adults with low education: randomised controlled trial. BMJ 341:c5370 20978060 10.1136/bmj.c5370 PMC2965151

[40] Leighl NB, Shepherd HL, Butow PN, Clarke SJ, McJannett M, Beale PJ et al (2011) Supporting treatment decision making in advanced cancer: a randomized trial of a decision aid for patients with advanced colorectal cancer considering chemotherapy. J Clin Oncol 29(15):2077–2084 21483008 10.1200/JCO.2010.32.0754

[41] Lewis C, Pignone M, Schild L, Scott T, Winquist A, Rimer B et al (2010) Effectiveness of a patient and practice-level colorectal cancer screening intervention in health plan members: design and baseline findings of the CHOICE trial. Cancer 116(7):1664–1673 20143439 10.1002/cncr.24962 PMC2938956

[42] Green MJ, Biesecker BB, McInerney AM, Mauger D, Fost N (2001) An interactive computer program can effectively educate patients about genetic testing for breast cancer susceptibility. Am J Med Genet 103(1):16–23 11562929 10.1002/ajmg.1500

[43] Mathieu E, Barratt AL, McGeechan K, Davey HM, Howard K, Houssami N (2010) Helping women make choices about mammography screening: an online randomized trial of a decision aid for 40-year‐old women. Patient Educ Couns 81(1):63–72 20149953 10.1016/j.pec.2010.01.001

