# Supplementary material for: Shared decision-making interventions in neuro-oncology practice: a systematic review
Source: J Neurooncol. 2025 Jun 23;175(2):471–9. doi: 10.1007/s11060-025-05141-7 (PMC12420689; doi:10.1007/s11060-025-05141-7)
Supplement: Supplementary file 1 — Supplementary Material 1 [file 11060_2025_5141_MOESM1_ESM.docx]

**SUPPLEMENTAL MATERIALS**

**Search Strategies**

Ovid MEDLINE(R) and Epub Ahead of Print, In-Process, In-Data-Review & Other Non-Indexed Citations, Daily and Versions <1946 to August 22, 2024>

1 decision making/ or consensus/ or decision making, shared/ or decision support techniques/ 151585

2 ("decision support" or "SDM model*" or "decision aid*" or "decision tool*" or "decision participat*" or "decision making" or consensus).ti,ab,kw,kf. 463217

3 ("decision making" adj2 (collaborat* or share* or sharing or joint or assist* or patient*)).ti,ab,kw,kf. 27980

4 1 or 2 or 3 540334

5 (Challeng* or intervention* or barrier* or facilitat* or policy or policies or guideline* or guidance or "organisational culture" or "organizational culture" or attitude*).ti,ab,kw,kf. 4536907

6 Policy/ or Organizational Policy/ or Practice Guideline/ or Guideline/ or organizational culture/ 82017

7 5 or 6 4572116

8 (neurosurg* or "neuro-surg*" or neuroncolog* or "neuro-oncolog*").ti,ab,kw,kf. 73422

9 Neurosurgery/ or neurosurgical procedures/ 51095

10 8 or 9 103340

11 4 and 7 and 10 1614

Embase <1974 to 2024 August 22>

1 ("decision support" or "SDM model*" or "decision aid*" or "decision tool*" or "decision participat*" or "decision making" or consensus).ti,ab. 616419

2 ("decision making" adj2 (collaborat* or share* or sharing or joint or assist* or patient*)).ti,ab. 38352

3 decision support system/ or *clinical decision support system/ or *shared decision making/ or *decision making/ or *consensus/ or *medical decision making/ or *patient decision making/ 135629

4 1 or 2 or 3 675858

5 (Challeng* or intervention* or barrier* or facilitat* or policy or policies or guideline* or guidance or "organisational culture" or "organizational culture" or attitude*).ti,ab. 5852749

6 *policy/ or *practice guideline/ or *organizational culture/ 128188

7 5 or 6 5888392

8 *neurosurgery/ 32661

9 (neurosurg* or "neuro-surg*" or neuroncolog* or "neuro-oncolog*").ti,ab. 97845

10 8 or 9 112899

11 4 and 7 and 10 2104

Global Health (via Ebscohost)

TI ( "decision support" or "SDM model*" or "decision aid*" or "decision tool*" or "decision participat*" or "decision making" or consensus ) OR AB ( "decision support" or "SDM model*" or "decision aid*" or "decision tool*" or "decision participat*" or "decision making" or consensus ) OR TI ( ("decision making" N2 (collaborat* or share* or sharing or joint or assist* or patient*)) ) OR AB ( ("decision making" N2 (collaborat* or share* or sharing or joint or assist* or patient*)) ) OR ((DE "decision support systems") OR (DE "consensus")) OR (DE "decision making")

AND

TI ( Challeng* or intervention* or barrier* or facilitat* or policy or policies or guideline* or guidance or "organisational culture" or "organizational culture" or attitude* ) OR AB ( Challeng* or intervention* or barrier* or facilitat* or policy or policies or guideline* or guidance or "organisational culture" or "organizational culture" or attitude* ) OR (((((DE "policy") OR (DE "intervention")) OR (DE "barriers")) OR (DE "guidelines")) OR (DE "guidance")) OR (DE "attitudes")

AND

TI (neurosurg* or “neuro-surg*” or neuroncolog* or “neuro-oncolog*”) OR AB (neurosurg* or “neuro-surg*” or neuroncolog* or “neuro-oncolog*”)

Cinahl (via Ebscohost)

TI ( "decision support" or "SDM model*" or "decision aid*" or "decision tool*" or "decision participat*" or "decision making" or consensus ) OR AB ( "decision support" or "SDM model*" or "decision aid*" or "decision tool*" or "decision participat*" or "decision making" or consensus ) OR TI ( ("decision making" N2 (collaborat* or share* or sharing or joint or assist* or patient*)) ) OR AB ( ("decision making" N2 (collaborat* or share* or sharing or joint or assist* or patient*)) ) OR (MH "Decision Support Techniques") OR (MH "Consensus") OR (MH "Decision Making") OR (MH "Decision Making, Shared") OR (MH "Decision Making, Patient") OR (MH "Decision Making, Clinical")

AND

TI ( Challeng* or intervention* or barrier* or facilitat* or policy or policies or guideline* or guidance or "organisational culture" or "organizational culture" or attitude* ) OR AB ( Challeng* or intervention* or barrier* or facilitat* or policy or policies or guideline* or guidance or "organisational culture" or "organizational culture" or attitude* ) OR (MH "Organizational Policies") OR (MH "Hospital Policies") OR (MH "Organizational Culture") OR (MH "Practice Guidelines") OR (MH "Attitude")

AND

TI (neurosurg* or “neuro-surg*” or neuroncolog* or “neuro-oncolog*”) OR AB (neurosurg* or “neuro-surg*” or neuroncolog* or “neuro-oncolog*”) OR (MH "Neurosurgery")

# Web of Science Search Strategy (v0.1)

# Database: Web of Science Core Collection

# Entitlements:

- WOS.IC: 1993 to 2024

- WOS.CCR: 1985 to 2024

- WOS.SCI: 1900 to 2024

- WOS.AHCI: 1975 to 2024

- WOS.BHCI: 2008 to 2024

- WOS.BSCI: 2008 to 2024

- WOS.ESCI: 2019 to 2024

- WOS.ISTP: 1990 to 2024

- WOS.SSCI: 1956 to 2024

- WOS.ISSHP: 1990 to 2024

# Searches:

1: TS=("decision support" or "SDM model*" or "decision aid*" or "decision tool*" or "decision participat*" or "decision making" or consensus) Date Run: Thu May 02 2024 11:41:32 GMT+0100 (British Summer Time) Results: 967998

2: TS=(("decision making" Near/2 (collaborat* or share* or sharing or joint or assist* or patient*))) Date Run: Thu May 02 2024 11:41:52 GMT+0100 (British Summer Time) Results: 39852

3: #2 OR #1 Date Run: Thu May 02 2024 11:41:58 GMT+0100 (British Summer Time) Results: 967998

4: TS=(Challeng* or intervention* or barrier* or facilitat* or policy or policies or guideline* or guidance or "organisational culture" or "organizational culture" or attitude*) Date Run: Thu May 02 2024 11:42:18 GMT+0100 (British Summer Time) Results: 8253976

5: TS=(neurosurg* or “neuro-surg*” or neuroncolog* or “neuro-oncolog*”) Date Run: Thu May 02 2024 11:42:37 GMT+0100 (British Summer Time) Results: 61817

6: #5 AND #4 AND #3 Date Run: Thu May 02 2024 11:42:45 GMT+0100 (British Summer Time) Results: 1180

Scopus

( ( TITLE-ABS-KEY ( "decision support" OR "SDM model*" OR "decision aid*" OR "decision tool*" OR "decision participat*" OR "decision making" OR consensus ) ) OR ( TITLE-ABS-KEY ( ( "decision making" W/2 ( collaborat* OR share* OR sharing OR joint OR assist* OR patient* ) ) ) ) ) AND ( TITLE-ABS-KEY ( challeng* OR intervention* OR barrier* OR facilitat* OR policy OR policies OR guideline* OR guidance OR "organisational culture" OR "organizational culture" OR attitude* ) ) AND ( TITLE-ABS-KEY ( neurosurg* OR "neuro-surg*" OR neuroncolog* OR "neuro-oncolog*" ) )

Global Index Medicus

Title, abstract, subject fields: (neurosurgery) AND (shared decision making)

Overton

Keyword field: "shared decision making" AND neurosurgery

Results

| Database | Results 23/08/2024 |
| --- | --- |
| Medline | 1614 |
| Embase | 2014 |
| Global Health | 31 |
| Cinahl | 109 |
| Web of Science | 1379 |
| Scopus | 3166 |
| Global Index Medicus | 0 |
| Overton | 690 |
| After deduplication | 4889 |
